# Modulatory role of blackberry fruit standardized extract on brain redox status and insulin signaling in type 2 diabetic rats

**DOI:** 10.1007/s11033-026-11735-9

**Published:** 2026-04-03

**Authors:** Imtiaz Ahmad, Julia Eisenhardt de Mello, Larissa Menezes da Silveira, Mayara Sandrielly Soares de Aguiar, Marcia Vizzotto, William Borges Domingues, Lucas Petitemberte de Souza, Vinicius Farias Campos, Rejane Giacomelli Tavares, Roselia Maria Spanevello, Francieli Moro Stefanello

**Affiliations:** 1 Laboratório de Biomarcadores, Centro de Ciências Químicas, Farmacêuticas e de Alimentos, Universidade Federal de Pelotas, Pelotas, RS Brazil; 2 Laboratório de Neuroquímica, Inflamação e Câncer, Centro de Ciências Químicas, Farmacêuticas e de Alimentos, Universidade Federal de Pelotas, Pelotas, RS Brazil; 3 Empresa Brasileira de Pesquisa Agropecuária, Centro de Pesquisa Agropecuária de Clima Temperado, Pelotas, RS Brazil; 4 Laboratório de Genômica Estrutural, Programa de Pós-Graduação em Biotecnologia, Centro de Desenvolvimento Tecnológico, Universidade Federal de Pelotas, Pelotas, RS Brazil

**Keywords:** Blackberry fruit, Metformin, Diabetes, Reactive species, Insulin signaling

## Abstract

**Background:**

This study explored the effect of blackberry fruit extract (BFE) and metformin (Met) on biochemical markers, redox parameters, and the downstream insulin signaling pathway in a high-fat diet (HFD) + streptozotocin (STZ) induced Type 2 Diabetic Rat model.

**Methods:**

The identification and quantification of cyanidin-3-glucoside by HPLC in the BFE revealed a concentration of 28.33 ± 0.05 mg/g of dry extract. For in vivo study, Type 2 Diabetes Mellitus (T2DM) was induced in rats by feeding a HFD for 3 weeks, followed by a single intraperitoneal dose of STZ on day 21. Animals were divided into 4 groups: I – Control, II – T2DM, III – T2DM plus Met (250 mg/kg) and IV – T2DM plus BFE (200 mg/kg). After STZ administration, the animals underwent a glucose tolerance test. Biochemical and molecular analyses were performed in serum and brain tissues.

**Results:**

BFE and Met improved diabetic parameters, mitigating hyperglycemia, dyslipidemia, and inflammation by lowering serum glucose, total cholesterol, triglycerides, very low-density lipoprotein, interleukin-6, adenosine deaminase activity, and reactive species levels, while restoring high-density lipoprotein and paraoxonase-1 activity in blood. Both treatments reduced oxidative stress in the cerebral cortex by lowering reactive species, nitrite, and thiobarbituric acid reactive substances levels and increasing sulfhydryl groups, superoxide dismutase and catalase activity. Also, BFE upregulated phosphatidylinositol 3-kinase, insulin receptor substrate-1, forkhead box O-3a in the cerebral cortex.

**Conclusion:**

These findings highlight the potential of BFE as a promising natural therapeutic agent for T2DM management, demonstrating efficacy comparable to Met in addressing metabolic and oxidative impairments.

**Graphical abstract:**

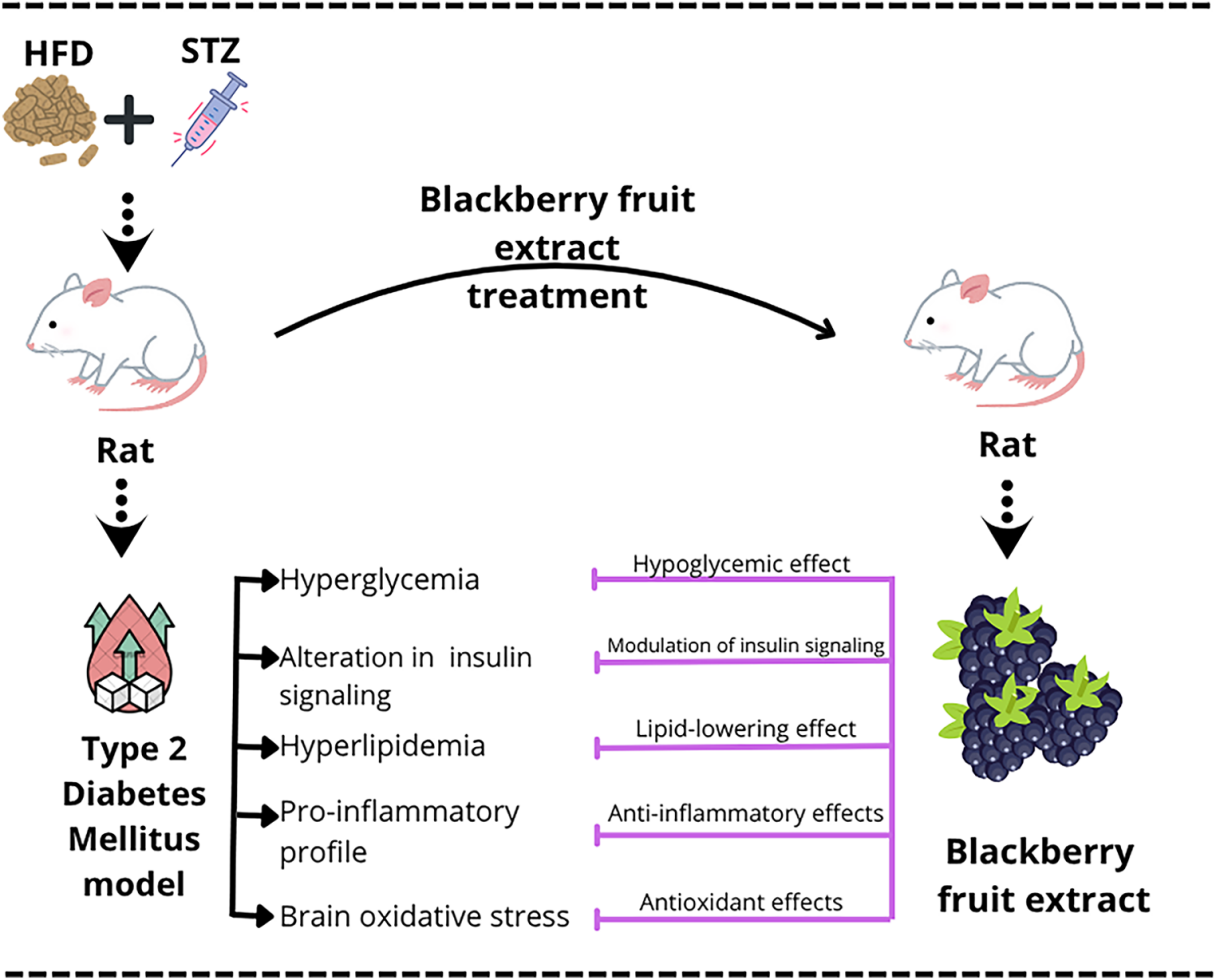

## Introduction

Chronic non-communicable diseases are today the main causes of death worldwide. Among these, type 2 diabetes mellitus (T2DM) is included, which can be determined mainly through hyperglycemia and insulin resistance [1]. T2DM currently affects about 463 million people worldwide, the prospects for the future are alarming, and it is expected that around 578 million people will have T2DM in 2030 [2].

It is linked with hyperglycemia and long-term complications that affect different organs like the kidneys, blood vessels, brain, and eyes [3]. Hyperglycemia results from insufficient insulin secretion or cellular resistance to insulin or both and is associated with disturbances in protein, lipid, and carbohydrate metabolism [3]. In T2DM, hyperglycemia is a major driver of oxidative stress and alterations in both lipid and glucose metabolism. Therefore, controlling blood glucose levels and oxidative stress is essential for reducing the progression of peripheral and neurological complications [4].

Phosphatidylinositol 3-kinase (PI3K)/serine/threonine kinase protein B (AKT) is the downstream insulin signaling pathway. It plays an important role in cell proliferation, differentiation, and glucose metabolism, and is closely linked with insulin resistance-related T2DM [5]. The binding of insulin to its receptor activates PI3K, which leads to AKT activation. Then, AKT activates the downstream effectors, as glycogen synthase kinase 3-β (GSK3β) and forkhead box O (FOXO) protein, thereby modulating glucose homeostasis [6]. It is reported that drugs enhancing this pathway can significantly improve insulin sensitivity and contribute to the management of T2DM [6]. Studies suggest that natural compounds derived from medicinal plants can effectively manage metabolic syndromes such as T2DM, often with minimal or no side effects [7, 8].

Blackberry (*Rubus*sp) belongs to Rosaceae family and is native to South America, North America, Asia, Europe, and Africa [9]. It is a rich source of anthocyanins, ellagitannins, and other key phenolic compounds which serve an important role in many diseases such as diabetes, obesity, cancer and coronary heart disease [10]. Several studies reported the association of blackberry with different health benefits in metabolic disorders, cardiovascular disease, cancer, Alzheimer’s, and depression [11–13]. However, despite this broad pharmacological potential, the specific effects of blackberry particularly its anthocyanin-rich extract on brain insulin signaling and oxidative stress in the context of T2DM remain largely unexplored. While previous research has demonstrated the systemic antidiabetic and antioxidant actions of plant-based compounds, few have investigated their impact on central insulin signaling pathways. The brain is now recognized as an insulin-sensitive organ, and brain insulin resistance is increasingly linked to neurodegeneration and cognitive impairment in T2DM [11]. This study uniquely evaluated the effect of blackberry fruit extract (BFE) on neuronal insulin signaling and oxidative stress markers in diabetic brain regions, extending the known therapeutic potential of blackberry to include the neurological complications of diabetes [12].

Therefore, the present study aims to characterize BFE and to evaluate its modulatory effects on selected biochemical and molecular parameters in serum and brain tissues. We hypothesize that these parameters mediate the biological actions of BFE in rats with T2DM induced by a (HFD) combined with streptozotocin (STZ).

## Materials and methods

### Extract preparation

The genotypes of *Rubus*sp*.* (TUPY, officially known as BRS TUPY), belonging to the plant breeding program and the Active Germplasm Bank at *Embrapa Clima Temperado*, RS, Brazil, were used. It was bred from a hybrid cross between the ‘Uruguai’ clone and the ‘Comanche’ cultivar. The fruit was collected and refrigerated at − 20 °C to preserve the integrity of the sample and protect from light. For extract preparation, the frozen blackberry fruits were sliced, and 50 g of the fruits were taken. Then, 150 mL 70% alcohol (pH 1.0) was added and placed in an ultrasound bath for 30 min [13]. The pH of the solution was adjusted to 1.0 because anthocyanins are highly sensitive to pH, and their extraction and stability are greatest under strongly acidic conditions. After, filter it with cotton. Again 150 mL alcohol was added and kept in an ultrasound bath for 30 min, repeating this step four times. After sonication and filtration, the pH of the solution was adjusted to 4, and then placed in a freezer for 24 h. Subsequently, the solvent completely evaporated using rotary evaporation to get the crude extract. Finally, the extract was lyophilized and stored in an ultrafreezer at – 80 °C [13].

### Identification and quantification of cyanidin-3-glucoside content

Chromatographic analyses were performed by Agilent^®^ 1260 Infinity II HPLC system equipped with a Zorbax^®^ Eclipse Plus C18 column, maintained at 40 °C. The autosampler was kept at 5 °C. The mobile phase consisted of ultrapure water with 0.1% formic acid (A) and acetonitrile with 0.1% formic acid (B), with a flow rate of 0.4 mL/min and an injection volume of 10 µL. A gradient elution was applied, ranging from 5% to 60% B over 21 min, followed by a re-equilibration to initial conditions. The total run time was 28 min. Detection was carried out using a diode array detector (DAD) at 520 nm. Samples were previously diluted in ultrapure water to a final concentration of 0.125 mg/mL. The acquisition method was pre-standardized for the identification of the target compound, cyanidin-3-glucoside, which showed an average retention time of 5.78 min.

### Animals and ethical aspects

Sixty days old (based on previous study) [14] male Wistar rats were provided and maintained by the Central Animal House of the Federal University of Pelotas (RS, Brazil) at 22 ± 1 °C under a 12:12 h light-dark cycle. Food and water were offered *ad libitum*. All animal procedures were consented by Institutional Ethics Commission (CEEA 5747/2015) and conducted according to the “Guide for the Care and Use of Laboratory Animals” (US National Institutes of Health publication no. 85–23, 1996). The experimental design complied with institutional and international ethical guidelines, including principles of Replacement, Reduction and Refinement (3Rs), and followed the ARRIVE recommendations. All efforts were made to minimize the number of animals used and reduce discomfort or suffering.

### Experimental design

The experimental model of T2DM was induced by the association of HFD and STZ administration. Rats with fasting blood glucose (FBG) > 250 mg/dL were considered diabetic. Oral glucose tolerance test (OGTT) was conducted, demonstrating impaired glucose clearance.

Twenty-eight Wistar rats were divided into four groups (7 in each group, based on previous study) [15]: I – Control (CT), II – T2DM, III – T2DM plus Met and IV – T2DM plus BFE. These animals received an HFD containing 45% lipids, 35% carbohydrates, and 20% protein from *Domeneghetti & Corrêa LTDA* (SP, Brazil) for 3 weeks followed by a single intraperitoneal (i.p.) dose of STZ (35 mg/kg) dissolved in 0.1 M sodium citrate solution [16]. Animals of group I received normal diet obtained from *Neovia Nutrição e Saúde Animal LTDA* (SP, Brazil) and 0.1 M sodium citrate solution by i.p route. Met (250 mg/kg) for group III [17] and BFE (200 mg/kg) for group IV [18] were administered intragastrically once a day simultaneously with the diet and continued throughout the experimental protocol period. Animals from group I and II received water in the same volume. At the end of the experimental protocol, the OGTT was performed in rats [13]. After that, animals were euthanized for serum and brain collection.

A dose of 200 mg/kg was selected based on previously published studies investigating the pharmacological and antioxidant effects of berry-derived extracts, which frequently employ this range to demonstrate efficacy without inducing toxicity [18]. Furthermore, our research group has previously shown that a 200 mg/kg dose provided greater protection against brain alterations induced by LPS [19] and ICV-STZ administration [18].

### Assessment of OGTT

After 72 h of STZ administration and 6 h of fasting, the OGTT was performed [13, 20]. A 50% glucose solution (2 g/kg body weight) was administered by gavage. Blood samples were collected at 0, 30, 60, and 120 min post-glucose administration through a small tail puncture. Blood glucose levels were measured using a glucometer (Accu-Chek Active, Roche Diagnostics^®^, USA). The area under the curve (AUC) over the time interval from 0 to 120 min was calculated using the linear trapezoidal rule method [20] as follows:

$$\:\frac{[C0+C30]\cdot[t30-t0]}{2}\:+\:\frac{[C30+C60]\cdot[t60-t30]}{2}\:+\:\frac{[C120+C60]\cdot[t120-t60]}{2}$$

C_0_, C_30_, C_60_, and C_120_ represent blood glucose concentrations (mg/dL) measured at 0, 30, 60 and 120 min post-glucose administration, respectively, and t_0_, t_30_, t_60_, and t_120_ denote the corresponding time points following glucose administration.

### Serum biochemical analysis

Glucose, total cholesterol, fractions (very low-density lipoprotein [VLDL], low-density lipoprotein [LDL], and high-density lipoprotein [HDL]), and triglyceride levels were determined using a colorimetric enzymatic method according to the manufacturer’s instructions (Labtest or BioCLin, MG, Brazil).

Paraoxonase-1 (PON1) activity was measured according to Browne et al. [21] and one unit of aryl esterase activity was considered equal to 1 mM of phenol formed per minute and expressed in U/mL.

Adenosine deaminase (ADA) activity was performed using the methodology previously described [22] in serum. The samples were submitted to the adenosine (Ado) reaction (21 mmol/L of Ado and pH 6.5) and incubated at 37 °C for 60 min. The specific activity of ADA was expressed in U/L. One unit (1U) of ADA was defined as the amount of enzyme required to release 1 mmol ammonia/min.

### IL-6 and insulin levels

IL-6 and insulin levels were measured in the serum using an ELISA kit employing recombinant rat antibodies (Sigma-Aldrich, St. Louis, MO, USA), according to the manufacturer’s instructions. Results were expressed as pg/mL for IL-6 and µIU/mL for insulin. Homeostatic model assessment for insulin resistance (HOMA-IR) was calculated as fasting insulin (µIU/mL) × fasting blood glucose (mmol/L)/22.5.

### Oxidative stress parameters

#### Tissue preparation

The cerebral cortex preparations and protein concentration were determined accordingly [18–20].

#### Reactive species (RS)

Reactive species (RS) levels were measured following the method described by Ali et al. [22]. Intracellular RS generation was assessed by the oxidation of 2’,7’-dichlorodihydrofluorescein diacetate (DCFH-DA) to the fluorescent compound dichlorofluorescein (DCF). After 30 min of incubation with DCFH-DA, the fluorescence intensity of DCF was measured at an excitation wavelength of 480 nm and an emission wavelength of 525 nm using a microplate reader (SpectraMax 190, Molecular Devices, San Jose, CA, USA). Results are expressed as µmol DCF/mg of protein.

#### Nitrite assay

Griess reaction was used for nitrite determination [23]. The brain homogenates sample reacted with Griess reagent (1% sulfanilamide, 1% naphthyl ethylenediamine chloride, and 25% H_3_PO_4_) for 10 min at room temperature. The absorbance was measured and then results were expressed as µM nitrite/mg of protein.

#### Total thiol (SH) content

Phosphate-buffered saline (PBS) with 1 mM ethylenediaminetetraacetic acid (EDTA) (pH 7.4) was added to the supernatant. The reaction was initiated by the addition of 10 mM 5,5′-dithio-bis-(2-nitrobenzoic acid) (DTNB) and incubated for 60 min at room temperature in the dark. Absorbance was then measured, and results were expressed as nmol TNB/mg of protein [24].

#### Thiobarbituric acid reactive substances (TBARS)

Trichloroacetic acid (TCA) 10% was added to the homogenates, which were centrifuged. Posteriorly the supernatant was mixed to 0.67% thiobarbituric acid (TBA) and incubated at 100 °C for 30 min. After this time, the plate was cooled and then read in a spectrophotometer. The results were expressed as nmol TBARS/mg of protein [25].

#### Antioxidant enzyme activity

Superoxide dismutase (SOD) activity was determined according to Misra and Fridovich [26]. The principle of this methodology lies in the fact that the autoxidation of adrenaline is highly dependent on oxygen. One unit of SOD is defined as the amount of SOD required to inhibit 50% of epinephrine autoxidation. Catalase (CAT) activity assay was measured according to Hamza and Hadwan [27] methodology, which is based on the disappearance of H_2_O_2_. One unit of the enzyme was defined as 1 mmol of H_2_O_2_ consumed per minute. SOD and CAT activity were reported as units/mg of protein.

### RNA extraction, cDNA synthesis and quantitative real-time polymerase chain reaction

Total mRNA was extracted from 50 to 100 mg of cerebral cortex tissue using TRIzol reagent (Invitrogen™, Carlsbad, USA) followed by DNase treatment with DNase I Amplification Grade (Invitrogen™, Carlsbad, USA). The total RNA isolated was quantified, and its purity was examined by spectrophotometer NanoVue (GE, Fairfield, CT, USA).

The cDNA synthesis was performed using a High-Capacity cDNA Reverse Transcription kit (AppliedBiosystems™, UK) according to the manufacturer’s protocol. For reverse transcription, 1 µg of total RNA was used in a reaction volume of 20 µL. The amplification was made with GoTaq^®^ qPCR Master Mix (Promega, Madison, WI) using the LightCycler^®^ 96 System (Roche Diagnostics, USA), and the sequence of primers used is indicated in Table 1. The qPCR conditions: 10 min at 95 °C to activate the hot-start Taq polymerase, followed by 35 cycles of denaturation for 15 s at 95 °C, primer annealing for 60 s at 60 °C, and extension for 30 s at 72 °C (fluorescence signals were detected at the end of every cycle). Baseline and threshold values were automatically set by the LightCycler^®^ 96 System software. The number of PCR cycles was defined as the Ct value, and each sample was analyzed in duplicate to obtain an average Ct for each sample. The 2^−ΔΔCT^ method was used to normalize the fold change in gene expressions by Schmittgen [28] using β‑actin as housekeeping gene.

**Table 1:** Forward and reverse primers used for quantitative real time polymerase chain reaction for amplification of GSK3β, NRF2, IRS1, FOXO3a, PI3K and β-actin

Primer name	Sequence	NCBI
GSK3β Forward	5’ GCCACAGCAGCCTCAGATAC 3’	NM_032080.1
GSK3β Reverse	5’ TGGGGCTGTTCAGGTAGAGT 3’	
NRF2 Forward	5’ CACATCCAGACAGACACCAGT 3’	NM_001399173.1
NRF2 Reverse	5’ CTACAAATGGGAATGTCTCTG 3’	
IRS1 Forward	5’ GGACGTCACAGCAGAATGAAG 3’	NM_012969.2
IRS1 Reverse	5’ GACGTGAGGTCCTGGTTGTG 3’	
FOXO3a Forward	5’ ACCCGCGAGTACAACCTTCT 3’	NM_001106395.1
FOXO3a Reverse	5’ ATACCCACCATCACACCCTGG 3’	
PI3K Forward	5’ TGGATATGAAGGGAGCCCCA 3’	NM_001371300.2
PI3K Reverse	5’ CATGCCCTAGGTGACCTGAC 3’	
β-actin Forward	5’ TGACAGGATGCAGAAGGAGA 3’	XM_039089807.1
β-actin Reverse	5’ GTACTTGCGCTCAGGAGGA 3’	

### Statistical analysis

Statistical analysis was performed by GraphPad Prism (version 8.0, San Diego, CA, USA). D’Agostino-Pearson normality test was employed to assess sample distribution. After, comparisons among the experimental groups were performed by one-way ANOVA or repeated measures ANOVA followed by Tukey multiple comparisons test (parametric data). The *p* < 0.05 values were considered significantly different.

## Results

### Phytochemical quantification of cyanidin-3-glucoside

The identification and quantification of cyanidin-3-glucoside in the extract were performed using high-performance liquid chromatography (HPLC). The analysis revealed a concentration of 28.33 ± 0.05 mg/g of dry extract.

### Effect of treatment with BFE and Met on OGTT

**Fig. 1:**
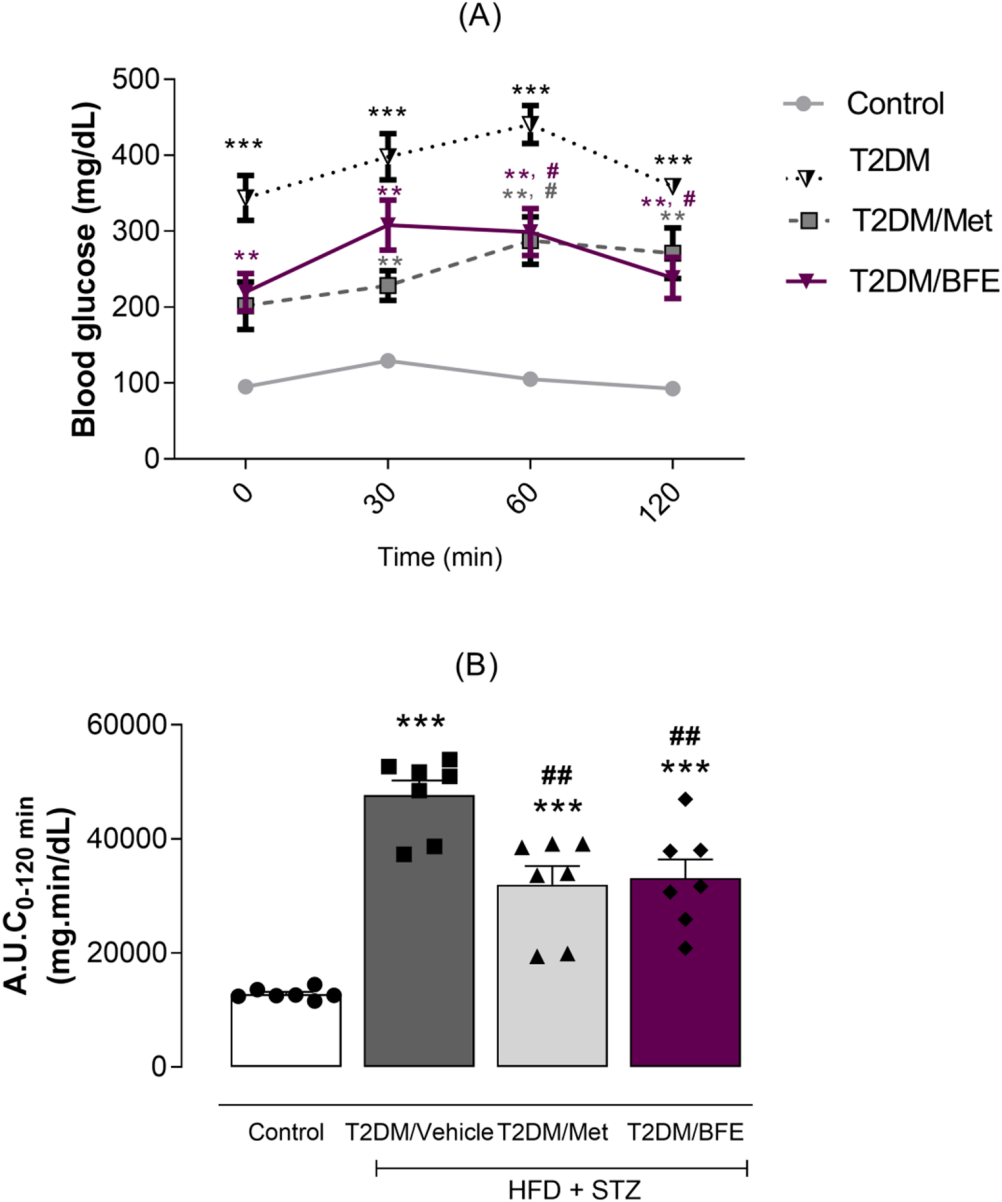
Oral glucose tolerance test demonstrating the variation in blood glucose during fasting and after 30, 60 and 120 min of glucose loading (1**A**) and the area under curve (AUC_0–120 min_) (1**B**). Data are expressed as mean ± S.E.M (*n* = 7). ^***^Represents *p* < 0.001 and ^**^represents *p* < 0.01 when compared to the control group. ^##^Represents *p* < 0.01 and ^#^ represents *p* < 0.05 when compared to the T2DM group. T2DM: Type 2 Diabetes Mellitus. BFE: Blackberry fruit extract. Repeated measures ANOVA and one-way ANOVA followed by Tukey *post hoc* test

HFD plus STZ resulted in a significant increase in the baseline blood glucose levels in all diabetic groups compared with CT group and the glucose level remained higher after loading glucose. In contrast, the interventions with Met and BFE showed a significant reduction in glucose levels at 60 and 120 min compared with untreated T2DM group (F_(9,72)_ = 3.604, *p* < 0.001, Fig. 1A).

To validate the significance of the observed changes, the AUC during the 120 min OGTT was calculated. The AUC was significantly elevated in the T2DM compared with the CT group. The treatments with Met or BFE significantly reduced the AUC, demonstrating protection against glucose intolerance (F_(3,24)_ = 28.87, *p* < 0.001, Fig. 1B).

### Effect of treatment with BFE and Met on serum the levels of glucose, cholesterol, TG, VLDL, HDL and PON1 activity

**Fig. 2:**
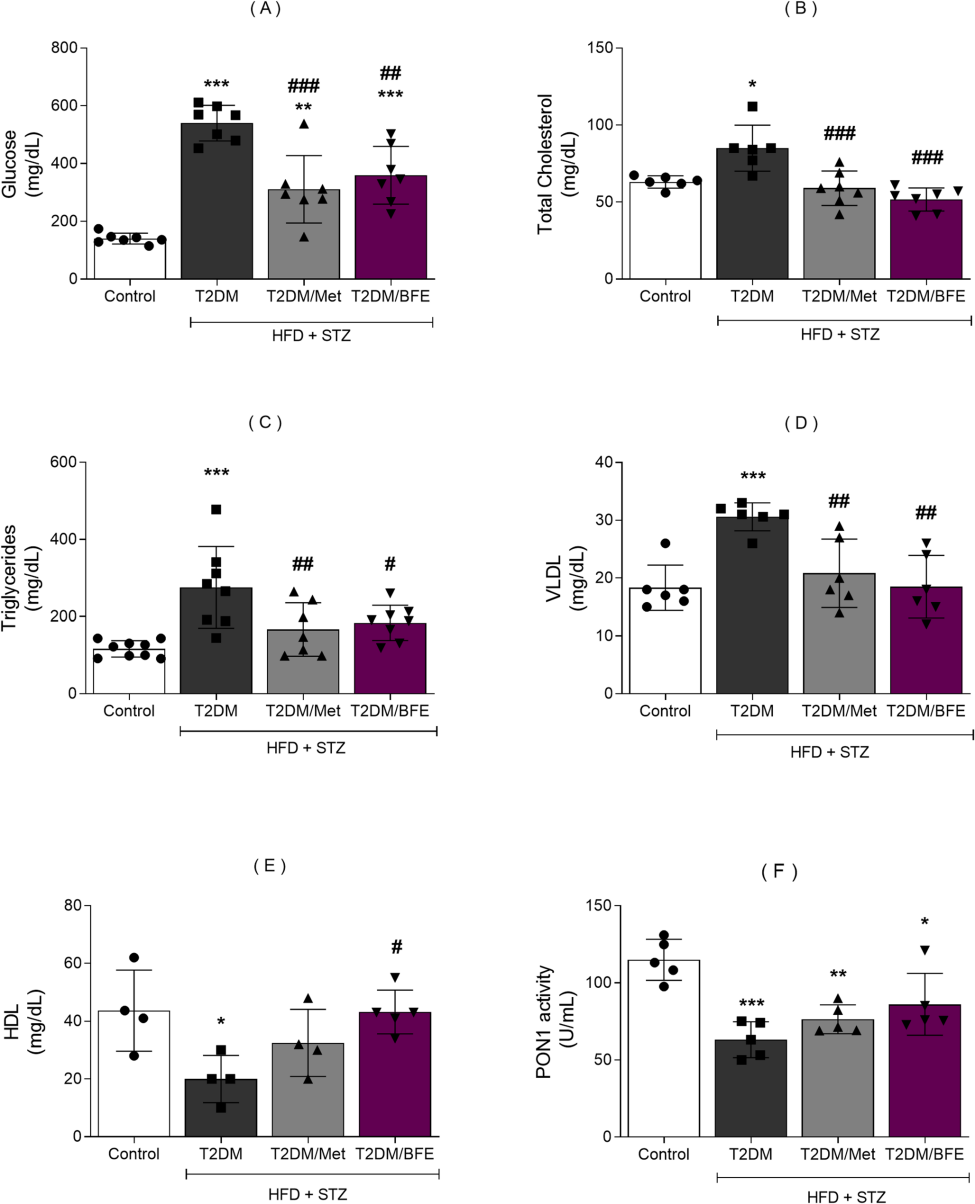
Effect of BFE and Met treatment on serum level of glucose (2**A**), cholesterol (2**B**), triglycerides (2**C**), VLDL (2**D**), HDL (2**E**) and PON1 activity (2**F**) in type 2 diabetic rats. Data are reported as mean ± S.D. (*n* = 4–7). ^***^represents *p* < 0.001, ^**^represents *p* < 0.01 and ^*^represents *p* < 0.05 when compared to the CT group. ^###^Represents *p* < 0.001, ^##^represents *p* < 0.01 and ^#^represents *p* < 0.05 when compared to the T2DM group. One way ANOVA followed by Tukey *post hoc* test. BFE: Blackberry fruit extract, T2DM: Type 2 Diabetes, Met: Metformin, LDL: Low density Lipoprotein, HDL: High Density Lipoprotein, HFD: High-fat diet and STZ: Streptozotocin

HFD + STZ increased the blood glucose level in all groups when compared with CT (*p* < 0.001). The treatment with BFE and Met reduced the blood glucose levels compared with T2DM group (*p* < 0.001) (F_(3, 24)_ = 27.30, Fig. 2A).

Cholesterol level was increased in the T2DM compared with CT group (*p* < 0.01), but both treatments (BFE and Met) (*p* < 0.001) were able to reduce the cholesterol total levels compared with T2DM group (F_(3, 22)_ = 12.44, Fig. 2B). Similarly, TG levels were increased in T2DM (*p* < 0.001) compared with CT group and both treatments (*p* < 0.001) reduced the TG levels compared with T2DM group (F_(3, 24)_ = 9.489, Fig. 2C). VLDL levels were also enhanced by HFD + STZ (*p <* 0.001) and both treatments were able to protect against this change (F_(3, 20)_ = 9.420, *p* > 0.05, Fig. 2D). Regarding HDL levels, a reduction was observed in the T2DM group and BFE was able to protect against this alteration (F_(3, 13)_ = 4.758, *p* < 0.05, Fig. 2E). PON1 activity was reduced in the T2DM compared with CT (*p* < 0.001), but neither treatment significantly enhanced the PON1 activity relative to T2DM group (*p* > 0.05), (F_(3, 16)_ = 12.05, Fig. 2F).

### Effect of treatment with BFE and Met on serum ADA and RS levels

**Fig. 3:**
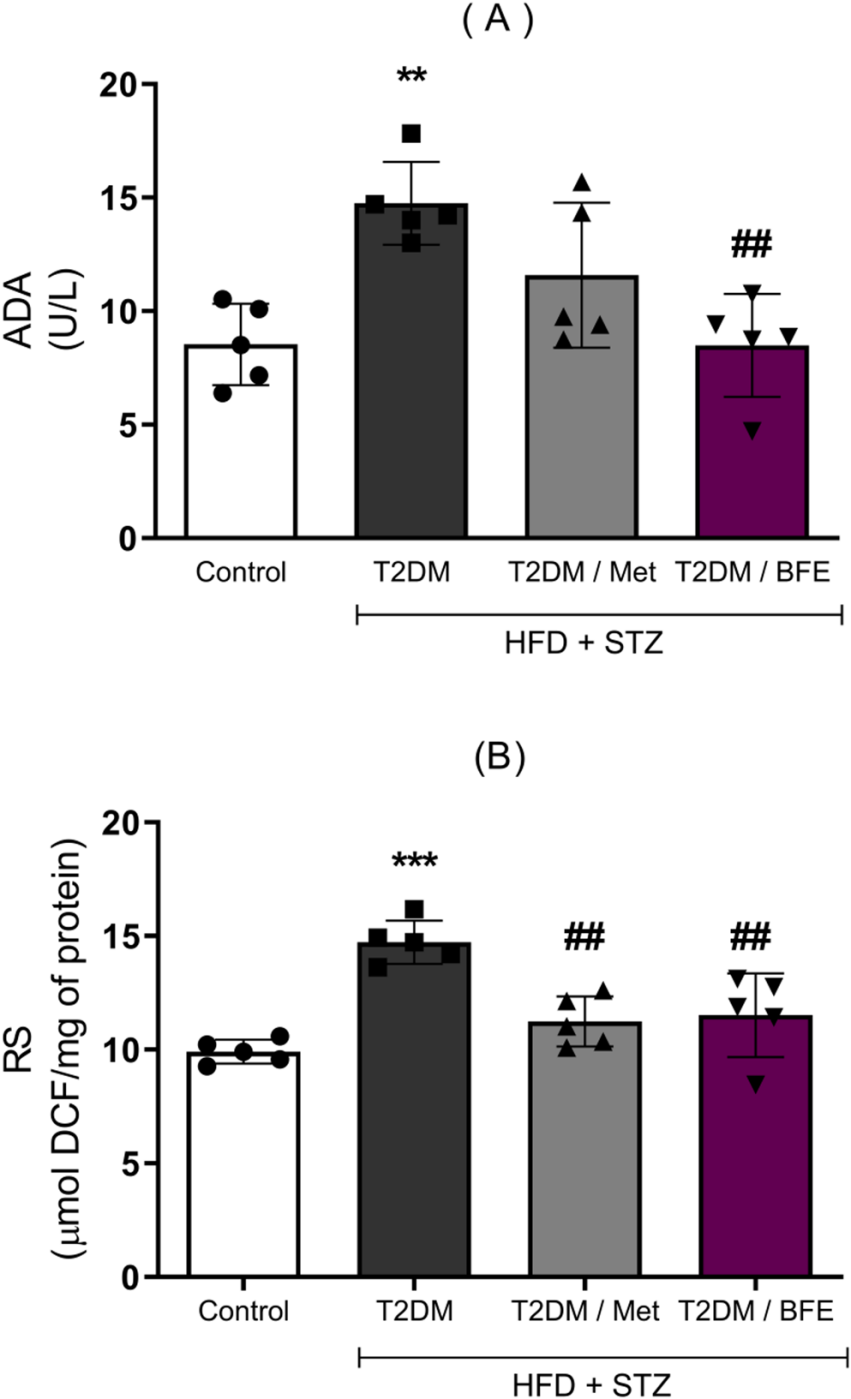
Effect of BFE and Met treatment on serum levels of ADA (3**A**) and RS (3**B**) in type 2 diabetic rats. Data are reported as mean ± S.D. (*n* = 5). ***represents *p* < 0.001, **represents *p* < 0.01 and *represents *p* < 0.05 when compared to the control group. ^##^Represents *p* < 0.01 and ^#^ represents *p* < 0.05 when compared to the T2DM group. One way ANOVA followed by Tukey *post hoc* test. ADA: Adenosine Deaminase, BFE: Blackberry fruit extract, RS: Reactive Species, T2DM: Type 2 Diabetes, Met: Metformin, HFD: High-fat diet and STZ: Streptozotocin

HFD + STZ increased ADA activity (*p* < 0.01) in serum and the treatment with BFE was able to protect against this change (*p* < 0.01) (F_(3, 16)_ = 8.149, Fig. 3A). Also, serum RS levels were increased (*p* < 0.001) by HFD + STZ in relation to CT, and the treatment with BFE (*p* < 0.01) and Met (*p* < 0.01) reduced these levels compared with T2DM group (F_(3, 16)_ = 14.51, Fig. 3B).

### Effect of treatment with BFE and Met on serum IL-6 and insulin levels

**Fig. 4:**
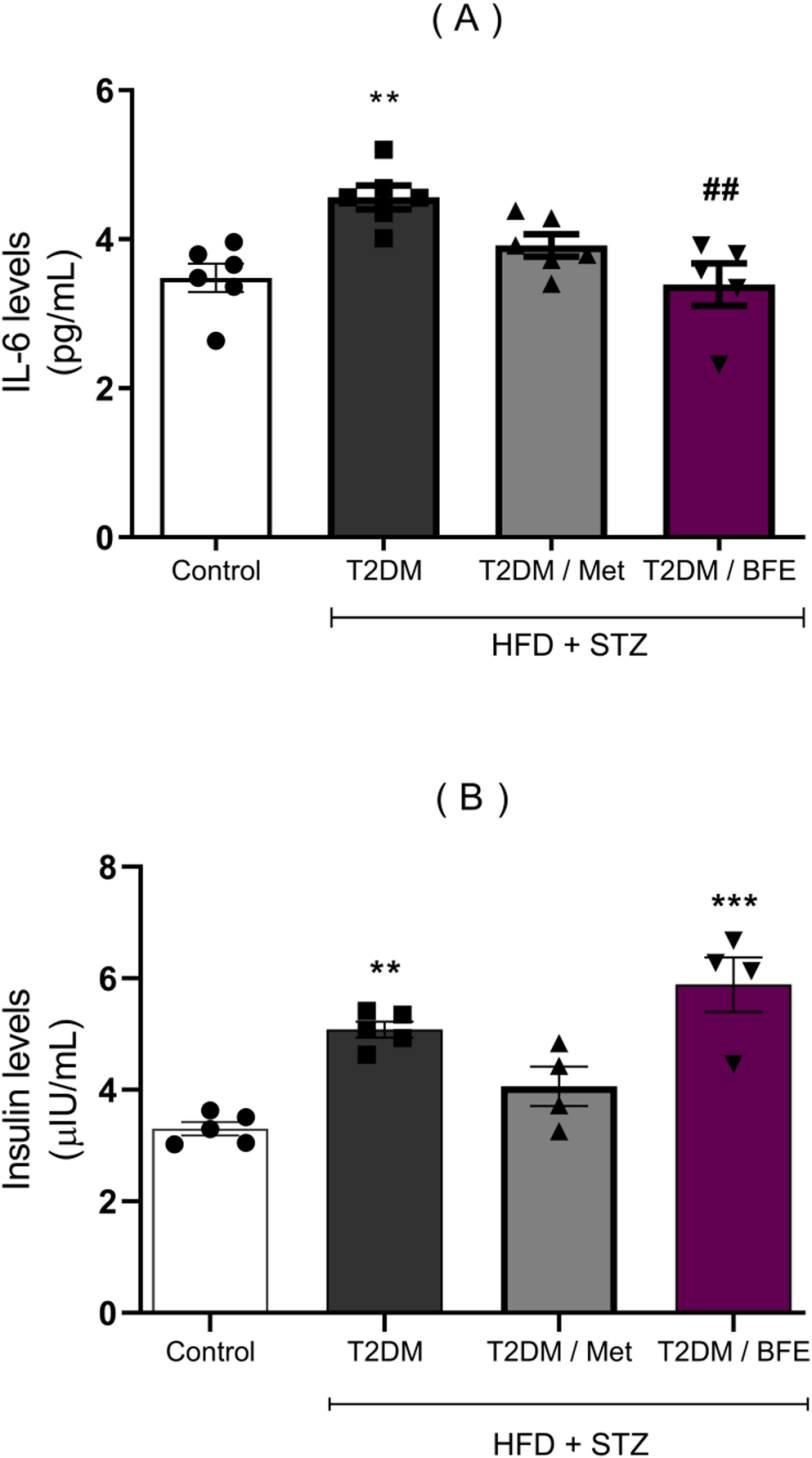
Effect of BFE and Met treatment on serum levels of IL 6 (4**A**) and insulin (4**B**) in type 2 diabetic rats. Data are reported as mean ± S.D. (*n* = 4–6). ***represents *p* < 0.001, **represents *p* < 0.01 when compared to the control group. ^##^represents *p* < 0.01 when compared to the T2DM group. One way ANOVA followed by Tukey *post hoc* test. BFE: Blackberry fruit extract, T2DM: Type 2 Diabetes, Met: Metformin, IL6: Interleukin 6, HFD: High-fat diet and STZ: Streptozotocin

HFD + STZ significantly increased IL-6 levels (*p* < 0.01) compared with the CT group. However, relative to the T2DM group, BFE treatment produced a significant reduction (*p* < 0.01) (F_(3, 19)_ = 7.487, Fig. 4A). HFD + STZ induced an increase in insulin level (*p* < 0.01) compared with CT. Both treatments did not reduce the insulin levels compared with T2DM group (*p* > 0.05) (F_(3, 14)_ = 15.95, Fig. 4B). However, the HOMA-IR value (CT: 1.14 ± 0.15; T2DM: 6.77 ± 0.77; Met: 3.11 ± 1.16; BFE: 5.15 ± 1.43) was increased in T2DM rats (*p* < 0.001) and decreased by Met (*p* < 0.001) and BFE (*p* < 0.05) treatments.

### Effect of treatment with BFE and Met on oxidative parameters in cerebral cortex

**Fig. 5:**
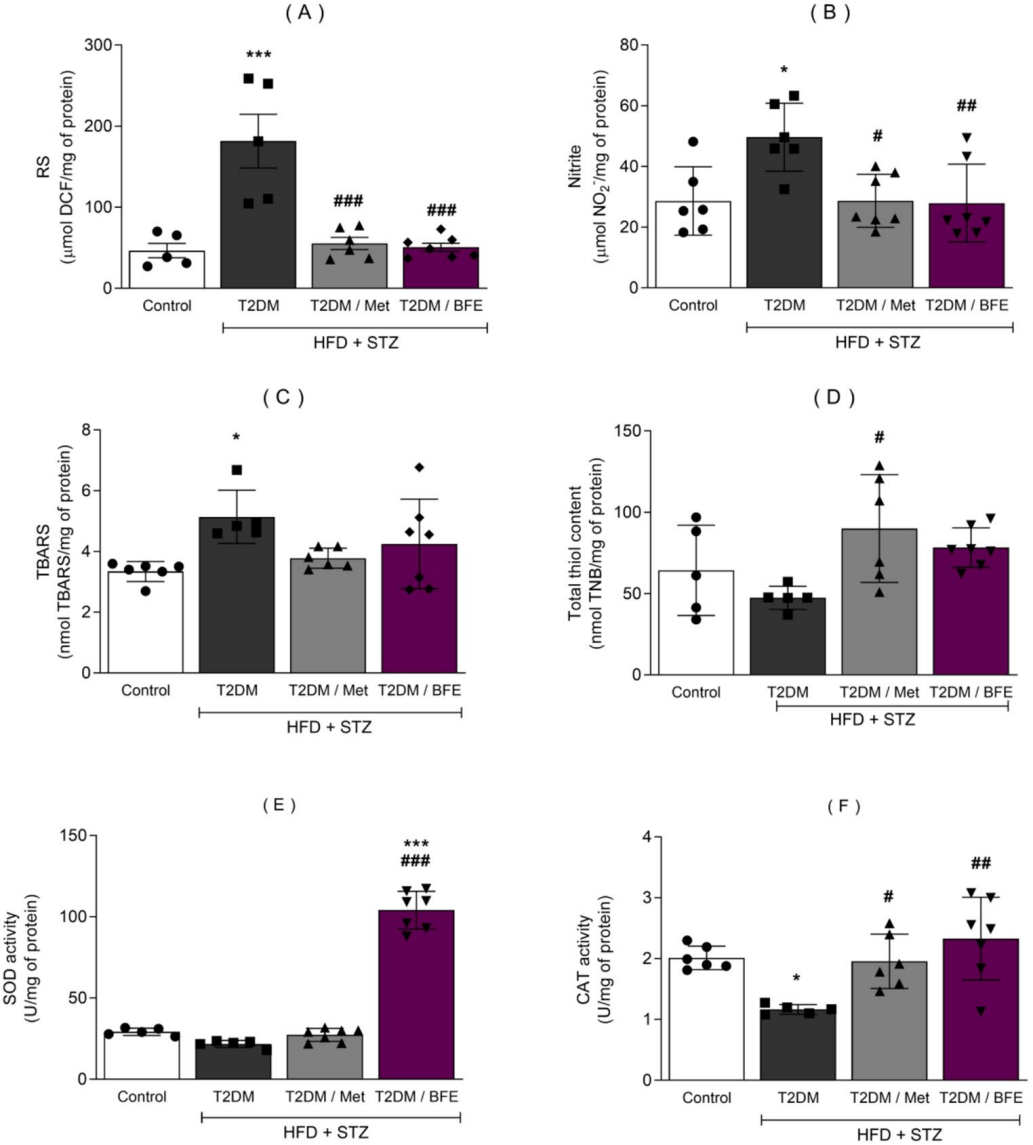
Effect of BFE and Met treatment on RS, nitrite, TBARS, SH level and SOD and CAT activity in the cerebral cortex of type 2 diabetic rats. Data are expressed as mean ± S.D. (5–7). ^*^Represents *p* < 0.05, ^**^represents *p* < 0.01 and ***represents *p* < 0.001 as compared to CT. ^#^represents *p* < 0.05, ^##^represents *p* < 0.01, ^###^represents *p* < 0.001 as compared to the T2DM group. One-way ANOVA followed by Tukey *post hoc* test. RS: Reactive Species, NO_2_^−^: Nitrite, TBARS: Thiobarbituric Acid Reactive Substances, SH: Total Thiol content, SOD: Superoxide Dismutase, CAT: Catalase, T2DM: Type 2 Diabetes, BFE: Blackberry fruit extract, Met: Metformin, HFD: High-fat diet and STZ: Streptozotocin

HFD + STZ induced T2DM increased the level of RS (F_(3, 19)_ = 16.08, *p* < 0.001, Fig. 5A), nitrite (F_(3, 22)_ = 5.620, *p* < 0.05, Fig. 5B) and TBARS (F_(3, 20)_ = 3.747, *p* < 0.05, Fig. 5D) compared with CT group. However, treatment with Met and BFE significantly decreased the level of RS (Met: *p* < 0.001, BFE: *p* < 0.001) and nitrite (Met: *p* < 0.05, BFE: *p* < 0.01). Moreover, no significant difference was shown by any group regarding SH (F_(3, 19)_ = 3.632, *p* > 0.05, Fig. 5C) level when compared with CT, while Met showed a significant difference of (*p* < 0.05) when compared with T2DM group. SOD activity was enhanced by BFE administration compared with T2DM group (F_(3, 20)_ = 212.0, *p* < 0.001, Fig. 5E). Also, HFD + STZ reduced CAT (F_(3, 20)_ = 6.841, *p* < 0.05, Fig. 5F) activity compared with CT and both treatments, Met (*p* < 0.05) and BFE (*p* < 0.01) were able to protect against this change.

### Effect of treatment with BFE and Met on mRNA expression of PI3K, IRS-1, GSK3β, NRF2 and FOXO3a in cerebral cortex

**Fig. 6:**
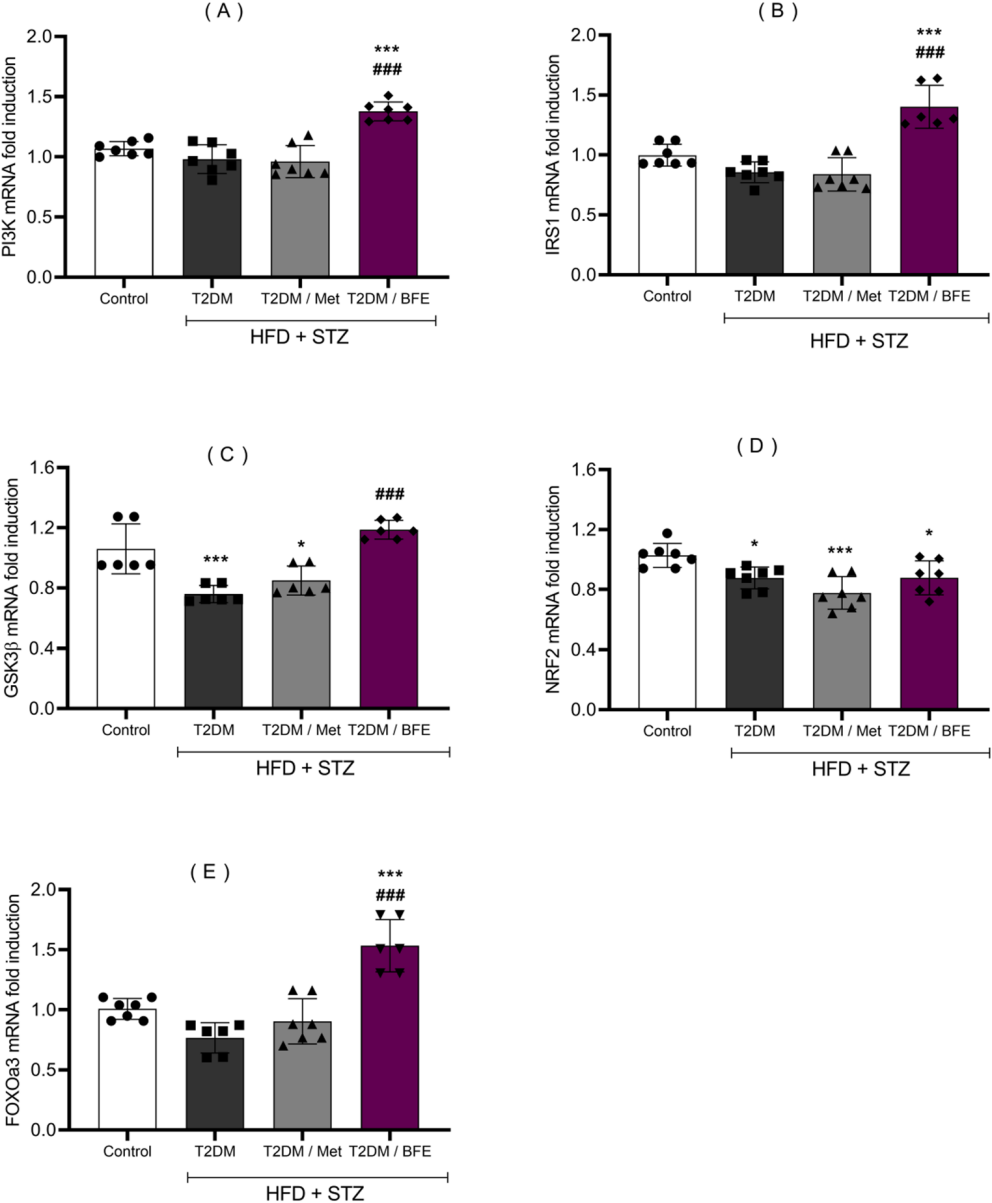
Evaluation of mRNA expression of phosphatidylinositol 3-kinase (PI3K) ( **6A**), insulin receptor substrate 1 (IRS1) ( 6**B**), glycogen synthase kinase 3-beta (GSK3β) (6**C**), nuclear factor erythroid 2-related factor 2 (NRF2) ( **6D**) and forkhead box O3 (FOXO3a) ( **6E**) in the cerebral cortex from animals submitted to the experimental model of T2DM. Data are expressed as mean ± S.D. (6–7). ^*^Represents *p* < 0.05 and *** represents *p* < 0.001 as compared to CT. ^###^Represents *p* < 0.001 as compared to the T2DM group. One-way ANOVA followed by Tukey *post hoc* test. T2DM: Type 2 Diabetes, BFE: Blackberry fruit extract, Met: Metformin, HFD: High-fat diet and STZ: Streptozotocin

PI3K (F_(3, 24)_ = 24.91, *p <* 0.001, Fig. 6A), IRS-1 (F_(3, 23)_ = 26.75, *p <* 0.001, Fig. 6B) and FOXO3a (F_(3, 22)_ = 26.10, *p <* 0.001, Fig. 6E) were increased in the group treated with BFE in relation to CT and T2DM groups. Also, GSK3β (F_(3, 20)_ = 20.71, *p* < 0.001, Fig. 6C) and NRF2 (F_(3, 24)_ = 8.191, *p* < 0.001, Fig. 6D) expression was reduced in the HFD + STZ group compared with CT group and the treatment with BFE was able to protect against GSK3β (*p* < 0.001) expression change.

## Discussion

Blackberry fruits are rich in phenolic compounds with anthocyanins representing the most prevalent subgroup. In this study, HPLC was employed to identify and quantify the anthocyanin (cyanidin-3-glucoside), revealing a concentration of 28.33 ± 0.05 mg/g of dry extract, similar with previous studies [29]. This alignment reinforces the reliability of our results and highlights the significant presence of anthocyanins in BFE. Further, this study was designed to investigate the deed of BFE on serum biochemical parameters, oxidative stress parameters, and T2DM related genes in STZ-induced diabetic rats.

The rat model combining HFD and low-dose STZ effectively reproduces key features of T2DM, including insulin resistance, β-cell dysfunction, hyperglycemia, dyslipidemia, and oxidative stress, reflecting the multifactorial nature of the disease [30]. Through GLUT2, cells absorb STZ, which causes the alkylation of proteins and leads to cell apoptosis [30]. Regarding glycemic control, Met exerts its effect and inhibits gluconeogenesis and activates the uptake of peripheral use of glucose [31, 32]. HFD + STZ induction significantly increased fasting glucose levels; however, treatment with BFE or Met significantly reduced fasting blood glucose compared with the untreated T2DM group. Besides, glucose tolerance testing revealed a significant group-by-time interaction, with the T2DM group showing persistently elevated glucose concentrations throughout the test, thereby confirming glucose intolerance. Met and BFE treatments produced a significant improvement in glucose tolerance.

Majority of complications in T2DM come from dysregulation of glucose and, consequently, metabolism of lipids. In diabetic dyslipidemia, insulin secretion and action play a leading role [33]. Abnormalities in lipid profile include elevated levels of TG, total cholesterol, LDL, and low levels of HDL [34, 35], similar to those found in this study in animals with T2DM. Importantly, the treatment with Met and BFE protects against the changes induced by the T2DM model in the markers of the lipid dysregulation in the blood. These effects may result from multiple mechanisms, including the action of phenolic compounds that can inhibit cholesterol absorption and enhance lipoprotein metabolism [36]. Furthermore, they can elevate the flow of bile and increase the excretion of bile cholesterol and bile acids [37]. They can also block the enzyme 3-hydroxy-3-methylglutaryl-CoA reductase, thereby reducing cholesterol synthesis. Additionally, they may inhibit acyl-CoA: cholesterol acyltransferase, leading to low cholesterol esterification in the intestine and liver [37].

PON1 is an enzyme serving as an antioxidant mainly synthesized in the liver and linked with HDL [38]. When the activity of PON1 decreases, the level of HDL also decreases and increases the risk of cardiovascular disease [38]. Herein, the T2DM model causes hyperlipidemia and reduces the activity of PON1. In this case, both treatments were not able to protect PON1 activity. However, a study reported that Met (300 mg/kg) for four weeks significantly brings change in the decreased activity of PON1 without changing lipid marker [31].

ADA is an enzyme involved in adenosine degradation and inflammation, with elevated activity observed in diabetes and hyperlipidemia [39]. Here, the T2DM model induced an increase in the ADA activity in blood, and the treatment with BFE and Met protected against this change. The anti-inflammatory effect associated with the nucleoside adenosine is well established. Thus, preventing an increase in ADA activity is of utmost importance for maintaining adequate extracellular levels of adenosine and thereby preserving the anti-inflammatory effects mediated by the activation of adenosinergic receptors present in many cells of the immune system, organs and tissues [40].

STZ administration significantly increased the level of IL-6, but BFE treatment protected against this change. Also, STZ induced an increase in insulin levels. Insulin is a hormone secreted by pancreatic β cells that plays a key role in glucose homeostasis by suppressing its production and promoting its uptake. In the context of insulin resistance, β cells increase insulin secretion in an attempt to overcome this resistance, leading to hyperinsulinemia [41]. In T2DM, hepatic insulin clearance is reduced, contributing further to elevated circulating insulin levels. Notably, insulin exhibits both anti-inflammatory and pro-inflammatory properties, and its serum levels have been associated with tissue swelling and hepatic lobular inflammation [42].

Pro-inflammatory cytokines also affect β cell disease, a study reported that IL-6 is involved in β cell dysfunction, and a high level of IL-6 is predicted to develop T2DM and decrease insulin sensitivity in liver cells [42]. It has been reported that phenolic compounds, such as those present in blackberry (ellagitannin, flavonols, and phenolic acids), can reduce pro-inflammatory cytokines by inhibiting the NF-κB pathway, which prevents the expression of inflammatory mediators like IL-6 [43]. Additionally, they modulate the NRF2/KEAP1 pathway, increasing the expression of antioxidant and anti-inflammatory genes, and inhibit the activation of MAPKs (ERK, JNK, and p38), which are involved in inflammatory signaling. They also reduce the expression of the enzymes COX-2 and iNOS, decreasing the production of inflammatory prostaglandins and nitric oxide [44]. Furthermore, they can act directly on immune cells, such as macrophages and lymphocytes, promoting an anti-inflammatory profile. These mechanisms make phenolic compounds important allies in controlling chronic inflammation, contributing to the prevention of metabolic, cardiovascular, and neurodegenerative diseases [45].

Differential effects were also observed for IL-6 and insulin levels across treatments. The reduction in IL-6 observed with BFE, but not with Met, likely reflects distinct mechanisms of action. Met primarily exerts its effects through AMPK activation and modulation of glucose metabolism [28], whereas BFE may act through antioxidant and anti-inflammatory pathways, consistent with its polyphenolic composition [8–10]. This suggests that BFE could serve as a complementary therapy to conventional antidiabetic drugs. With respect to insulin, BFE-treated rats exhibited a non-significant upward trend, potentially reflecting compensatory pancreatic activity or inherent variability of the experimental model [46]. In contrast, the HOMA-IR value was reduced following BFE treatment, suggesting that this extract may improve insulin sensitivity. Similar findings have been reported for red fruit extract of *Eugenia uniflora* [47].

Drugs for T2DM with neuroprotective effects are essential, as the disease increases the risk of cognitive decline, dementia, and peripheral neuropathy. These complications arise from brain insulin resistance, which impairs synaptic plasticity, as well as from oxidative stress, chronic inflammation, hyperglycemia, and vascular dysfunction, all of which accelerate neuronal damage and neurodegenerative processes [48]. Notably, new T2DM medications, such as GLP-1 agonists and DPP-4 inhibitors, have been widely studied not only for their antidiabetic effects but also for their ability to protect the brain by improving insulin signaling, reducing inflammation, and preventing neuronal death, thereby helping to preserve cognitive function in T2DM patients [49]. Therefore, it is of great relevance to highlight the neuroprotective effects of BFE.

Long term hyperglycemia and dyslipidemia are linked with oxidative stress [50]. The overproduction of ROS and RNS can induce cell damage, and the imbalance between the production of these species and their removal can lead to oxidative stress [49]. The level of these species is modulated by antioxidant enzymes such as SOD and CAT, imbalance in these enzymes can also lead to disturbance. Moreover, oxidative stress can inactivate these enzymes and proteins [51].

Hyperglycemia is the main agent in the generation of RS, so it plays a key role in the development of various T2DM complications because they oxidize and damage different biomolecules such as DNA, proteins, and lipids and lead to cell dysfunction and apoptosis [52]. In the current study, diabetic rats exhibited high levels of RS, followed by a change in the activity of SOD and CAT enzymes, which could intensify the increase in RS. Consequently, oxidative damage to biomolecules was found in the present study, observed through the increase in lipid peroxidation. Both treatments, Met and BFE, prevented oxidative changes induced by the T2DM model.

Our findings provide additional insight into the systemic effects of diabetes and highlight the underexplored role of CNS involvement. The neuroprotective effects of BFE can be associated with the antioxidants mechanisms of the polyphenolic compounds (ellagitannin, flavonols, and phenolic acids) present in the blackberry, as: [53] (i) Neutralization of free radicals due to the presence of hydroxyl groups capable of donating electrons; (ii) Chelation of transition metals, such as iron and copper, reducing the formation of reactive species; (iii) Modulation of antioxidant enzymes, such as SOD, catalase, and GPx; (iv) Inhibition of pro-oxidant enzymes, such as NADPH oxidase and xanthine oxidase; (v) Protection of biomolecules by preventing the oxidation of lipids, proteins, and DNA; and (vi) Regulation of cellular signaling pathways, such as NRF2/KEAP1 and NF-κB, enhancing the antioxidant response and reducing inflammatory processes [45]. Thus, the synergistic effect of the compounds present in BFE ensures action on multiple fronts against oxidative stress.

BFE also significantly improved serum biochemical parameters related to glucose and lipid metabolism, indicating systemic metabolic modulation. Given that hyperglycaemia and dyslipidaemia contribute to oxidative stress and neuronal damage in diabetes, the neuroprotective effects observed may be partly secondary to these improvements. However, changes in brain oxidative stress markers also suggest a possible direct action of BFE on the brain. Blackberry is rich in polyphenols, including ellagitannins and phenolic acids, as well as flavonoids such as flavonols and anthocyanins (notably cyanidin-3-glucoside). These compounds have been shown in several studies to cross the blood-brain barrier [54]. These compounds have demonstrated antioxidants, anti-inflammatory, and neuroprotective effects in the CNS following oral administration in animal models. In comparison, the relatively modest effects of Met on certain oxidative and molecular parameters may be attributed to its primary mechanism of action, which predominantly targets hepatic gluconeogenesis and peripheral insulin sensitivity, with limited direct antioxidant or anti-inflammatory activity [55]. Regarding the neuroprotective outcomes observed, these effects are likely mediated, at least in part, by systemic metabolic improvements such as enhanced glycemic control, reduced oxidative stress, and improved lipid homeostasis. Nevertheless, it is also plausible that specific polyphenolic constituents of BFE may exert direct effects within the CNS, as some dietary polyphenols have been reported to cross the blood-brain barrier [56]. Together, these mechanisms may collectively contribute to the neuroprotective benefits observed in this study.

PI3K/AKT is the main downstream signaling pathway of insulin and plays important roles in cell survival, differentiation, and glucose metabolism [57]. The impairment of the insulin signaling pathway can disrupt energy balance and contribute to various pathological conditions [58]. A pivotal step in this pathway is insulin binding to its receptors, which triggers downstream signaling through multiple routes [59]. The PI3K/AKT pathway is especially important as it promotes the movement of glucose transporters to the cell membrane, helping to maintain blood glucose balance [59]. In our study the STZ induced diabetes in all treated animals and downregulated the levels of GSK3β and NRF2. The diabetic animals treated with *Rubus sp.* have shown significant upregulation in the level of PI3K, IRS-1, GSK3β and FOXO3a as compared to both diabetic and/or control. Thus, our findings confirm that the BFE notably improves the insulin downstream signaling pathway. In addition, these results are aligned with Li et al. [60] which demonstrate that the administration of HFD + STZ downregulates insulin signaling pathway genes, and after the treatment with konjac glucomannan, the expression of IR, IRS1, IRS2, and PI3K upregulated significantly in liver.

Although the STZ–induced diabetic rat model is a well-established experimental approach for investigating metabolic and molecular alterations associated with diabetes, it does not fully capture the progressive and multifactorial nature of human T2DM. While the model reliably reproduces key features such as hyperglycemia and related metabolic disturbances, STZ induces β-cell dysfunction with a relatively rapid onset of diabetes, and potential extra-pancreatic effects cannot be entirely excluded. Additionally, it is important to mention that male rats were used to minimize variability associated with hormonal fluctuations in female rats. Nevertheless, sex-specific differences in metabolic regulation remain an important consideration and should be addressed in future investigations.

## Conclusion

Our study demonstrated that the BFE significantly modulated biochemical parameters, oxidative stress parameters, and pro-inflammatory cytokines, as shown by improved SOD activity, CAT activity and upregulating the downstream insulin signaling pathway. Collectively, these findings suggest that BFE represents a promising candidate for the management of T2DM. However, further studies are required to establish the complete phytochemical profile of BFE, to evaluate oxidative stress parameters in the liver, and to investigate the expression of key metabolic genes in the liver, adipose tissue, and skeletal muscle. In addition, validation at the protein level using Western blotting is necessary to fully elucidate the underlying molecular mechanisms and interconnected pathways contributing to the antidiabetic effects of *Rubus* sp.

## Data Availability

The datasets generated during the current study are available from the corresponding author on reasonable request.

## References

[1] Ruze R, Li T, Zou X, Song J, Chen Y, Xu R, Yin X, Xu Q (2023) Obesity and type 2 diabetes mellitus: connections in epidemiology, pathogenesis, and treatments. Front Endocrinol 14:1161521. 10.3389/fendo.2023.1161521 10.3389/fendo.2023.1161521 PMC10161731 37152942

[2] Oraii A, Shafiee A, Jalali A, Alaeddini F, Saadat S, Masoudkabir F, Vasheghani-Farahani A, Heidari A, Sadeghian S, Boroumand M, Karimi AH, Franco O (2022) Prevalence, awareness, treatment, and control of type 2 diabetes mellitus among the adult residents of tehran: Tehran Cohort Study. BMC Endocr Disord 22:248. 10.1186/s12902-022-01161-w 36253738 10.1186/s12902-022-01161-w PMC9578278

[3] Ağgül AG, Gür F, Gülaboğlu M (2021) Streptozotocin-induced oxidative stress in rats: the protective role of olive leaf extract. Bull Korean Chem Soc 42:180–187. 10.1002/bkcs.12157

[4] Ziamajidi N, Abbasalipourkabir R, Behrouj H, Nasiri A, Lotfi F (2017) Effects of aqueous extract of *Allium sativum* on biochemical parameters and oxidative stress in STZ-and STZ+ niacinamide induced diabetes mellitus rats. Ann Res Rev Biol 15:1–8. 10.9734/ARRB/2017/34448

[5] Manning BD, Toker A (2017) AKT/PKB signaling: navigating the network. Cell 169:381–405. 10.1016/j.cell.2017.04.001 28431241 10.1016/j.cell.2017.04.001 PMC5546324

[6] Huang X, Liu G, Guo J, Su Z (2018) The PI3K/AKT pathway in obesity and type 2 diabetes. Int J Biol Sci 14:1483–1496. 10.7150/ijbs.27173 30263000 10.7150/ijbs.27173 PMC6158718

[7] Zanzabil KZ, Hossain MS, Hasan MK (2023) Diabetes mellitus management: an extensive review of 37 medicinal plants. Diabetology 4:186–234. 10.3390/diabetology4020019

[8] Awuchi CG (2022) Medicinal plants, bioactive compounds, and dietary therapies for treating type 1 and type 2 diabetes mellitus. Nat Drugs Plants 237. 10.5772/intechopen.96470

[9] Tatar M, Varedi M, Naghibalhossaini F (2022) Epigenetic Effects of Blackberry Extract on Human Colorectal Cancer Cells. Nutr Cancer 74:1446–1456. 10.1080/01635581.2021.1952454 34282673 10.1080/01635581.2021.1952454

[10] Bader UL, Ain H, Tufail T, Javed M, Tufail T, Arshad MU, Hussain M, Abdulaali SS (2022) Phytochemical profile and pro-healthy properties of berries. Int J Food Propert 25:1714–1735. 10.1080/10942912.2022.2096062

[11] Arnold SE, Arvanitakis Z, Macauley-Rambach SL, Koenig AM, Wang HY, Ahima RS, Craft S, Gandy S, Buettner C, Stoeckel LE, Holtzman DM, Nathan DM (2018) Brain insulin resistance in type 2 diabetes and Alzheimer disease: concepts and conundrums. Nat Rev Neurol 14:168–181. 10.1038/nrneurol.2017.185 29377010 10.1038/nrneurol.2017.185 PMC6098968

[12] Moreno UR, González-Sarrías A, Espín JC, Tomás-Barberán FA, Janes M, Cheng H, Finley J, Greenway F, Losso JN (2022) Effects of red raspberry polyphenols and metabolites on the biomarkers of inflammation and insulin resistance in type 2 diabetes: a pilot study. Food Funct 13:5166–5176. 10.1039/d1fo02090k 35421887 10.1039/d1fo02090k

[13] Cardoso JS, Cardoso TF, De Mello JE, De Aguiar MSS, Oliveira PS, Saraiva JT, Vizzotto M, Grecco FB, Lencina CL, Spanevello RM, Tavares RG, Stefanello FM (2023) Psidium cattleianum fruit extract prevents systemic alterations in an animal model of type 2 diabetes mellitus: comparison with metformin effects. Biomarkers 28:238–248. 10.1080/1354750X.2022.2163695 36576409 10.1080/1354750X.2022.2163695

[14] Tony SK, Hassan MS, Ismail HA, El-Naem GFA, Gazwi HS (2023) Effect of anthocyanin-rich blackberry juice on endoplasmic reticulum stress in streptozotocin-induced diabetic rats. Environ Sci Pollut Res 30:79067–79081. 10.1007/s11356-023-27827-z 10.1007/s11356-023-27827-z PMC10313550 37280499

[15] Tiwari V, Kamboj A, Sheoran B, Chaudhary E, Yadav M, Kumari A, Krishania M, Ali U, Tiwari A, Garg M, Bhatnagar A (2025) Anthocyanin-rich black wheat as a functional food for managing type 2 diabetes mellitus: A study on high fat diet-streptozotocin-induced diabetic rats. Food Funct 16:3273–3295. 10.1039/D4FO05065G 39688703 10.1039/d4fo05065g

[16] Singh S, Sharma R, Malhotra S, Vipin A, Kapila S, Kapila R (2017) Lactobacillus rhamnosus NCDC17 ameliorates type-2 diabetes by improving gut function, oxidative stress and inflammation in high-fat-diet fed and streptozotocin-treated rats. Benef Microbes 8:243–255. 10.3920/BM2016.0114 28008783 10.3920/BM2016.0090

[17] Jiao Y, Wang X, Jiang X, Kong F, Wang S, Yan C (2017) Antidiabetic effects of Morus alba fruit polysaccharides on high-fat diet- and streptozotocin-induced type 2 diabetes in rats. J Ethnopharmacol 199:119–127. 10.1016/j.jep.2017.01.036 28163112 10.1016/j.jep.2017.02.003

[18] de Mello JE, Teixeira FC, Dos Santos A, Luduvico K, De Aguiar MSS, Domingues WB, Campos VF, Tavares RG, Schneider A, Stefanello FM, Spanevello RM (2024) Treatment with blackberry extract and metformin in sporadic Alzheimer’s disease model: impact on memory, inflammation, redox status, phosphorylated tau protein and insulin signaling. Mol Neurobiol 61:7814–7829. 10.1007/s12035-024-04062-2 38430352 10.1007/s12035-024-04062-2

[19] Custódio SV, Piccoli RC, Goularte KCM, Simões WS, de Mello JE, de Souza AA, de Mattos Almeida IP, Barschak AG, Tavares RG, Stefanello FM, de Aguiar MSS, Spanevello RM (2025) Blackberry extract prevents lipopolysaccharide-induced depressive-like behavior in female mice: implications for redox status, inflammation, and brain enzymes. Nutr Neurosci 28:194–208. 10.1080/1028415X.2024.2363570 38861649 10.1080/1028415X.2024.2363570

[20] Piccoli RC, Simões WS, Custódio SV, Goularte KCM, Luduvico KP, de Mello JE, de Souza AA, Teixeira AC, da Costa DA, Barschak AG, Deniz BF, Almeida W, Pereira P, Nicolai M, Spanevello RM, Stefanello FM, Tavares RG, Palma ML (2024) Sustainable intervention: grape pomace flour ameliorates fasting glucose and mitigates streptozotocin-induced pancreatic damage in a type 2 diabetes animal model. Pharmaceuticals 17:1530 39598440 10.3390/ph17111530 PMC11597639

[21] Browne RW, Koury ST, Marion S, Wilding GE, Muti P, Trevisan M (2007) Accuracy and biological variation of human serum paraoxonase 1 activity and polymorphism (Q192R) by kinetic enzyme assay. Clin Chem 53:310–317. 10.1373/clinchem.2006.073056 17185369 10.1373/clinchem.2006.074559

[22] Giusti G, Galanti B (1984) Colorimetric method. Adenosine deaminase. Methods of enzymatic analysis 4:3.

[23] Stuehr DJ, Nathan CF (1989) Nitric oxide: a macrophage product responsible for cytostasis and respiratory inhibition in tumor target cells. J Exp Med 169:1543–1555. 10.1084/jem.169.5.1543 2497225 10.1084/jem.169.5.1543 PMC2189318

[24] Aksenov MY, Markesbery WR (2001) Changes in thiol content and expression of glutathione redox system genes in the hippocampus and cerebellum in Alzheimer’s disease. Neurosci Lett 302:141–145. 10.1016/s0304-3940(01)01636-6 11290407 10.1016/s0304-3940(01)01636-6

[25] Ohkawa H, Ohishi N, Yagi K (1979) Assay for lipid peroxides in animal tissues by thiobarbituric acid reaction. Anal Biochem 95:351–358. 10.1016/0003-2697(790738-3 36810 10.1016/0003-2697(79)90738-3

[26] Misra HP, Fridovich I (1972) The role of superoxide anion in the autoxidation of epinephrine and a simple assay for superoxide dismutase. J Biol Chem 247:3170–3175 4623845

[27] Hamza TA, Hadwan MH (2020) New spectrophotometric method for the assessment of catalase enzyme activity in biological tissues. Curr Anal Chem 16:1054–1062. 10.2174/1573411016666200116091238

[28] Schmittgen TD (2001) Real-time quantitative PCR. Methods 25(4):383–385 11846607 10.1006/meth.2001.1260

[29] Chaves VC, Soares MSP, Spohr L, Teixeira F, Vieira A, Constantino LS, Dal Pizzol F, Lencina CL, Spanevello RM, Freitas MP, Simões CMO, Reginatto FH, Stefanello FM (2020) Blackberry extract improves behavioral and neurochemical dysfunctions in a ketamine-induced rat model of mania. Neurosci Lett 714:134566. 10.1016/j.neulet.2019.134566 31698027 10.1016/j.neulet.2019.134566

[30] Duan J, Yang M, Liu Y, Xiao S, Zhang X (2022) Curcumin protects islet beta cells from streptozotocin induced type 2 diabetes mellitus injury via its antioxidative effects. Endokrynol Pol 73:942–946. 10.5603/EP.a2022.0070 35971926 10.5603/EP.a2022.0070

[31] Wójcicka G, Jamroz-Wiśniewska A, Czechowska G, Korolczuk A, Marciniak S, Bełtowski J (2016) The paraoxonase 1 (PON1), platelet-activating factor acetylohydrolase (PAF-AH) and dimethylarginine dimethylaminohydrolase (DDAH) activity in the metformin treated normal and diabetic rats. Eur J Pharmacol 789:187–194. 10.1016/j.ejphar.2016.07.034 27450486 10.1016/j.ejphar.2016.07.034

[32] Meng XM, Ma XX, Tian YL, Jiang Q, Wang LL, Shi R, Ding L, Pang SG (2017) Metformin improves the glucose and lipid metabolism via influencing the level of serum total bile acids in rats with streptozotocin-induced type 2 diabetes mellitus. Eur Rev Med Pharmacol Sci 21:2232–2237 28537659

[33] Alsubhi MI, Sherbini RT, Alkhalifah AH, Alnajjar WZ, Ali H, Alqarni SAA, Alsufyani SA, Alharbi RK, Alfaqih YH, Khuraybah SM (2025) Dyslipidemia in diabetes: understanding the role of insulin in lipid metabolism. J Health Sci 5:189–195. 10.52533/JOHS.2025.50602

[34] Monnier L, Colette C (2015) Postprandial and basal hyperglycaemia in type 2 diabetes: contributions to overall glucose exposure and diabetic complications. Diabetes Metab 41(6 Suppl 1):6S9-6S15. 10.1016/S1262-3636(16)30003-9 26774019 10.1016/S1262-3636(16)30003-9

[35] Pourfarjam Y, Rezagholizadeh L, Nowrouzi A, Meysamie A, Ghaseminejad S, Ziamajidi N, Norouzi D (2017) Effect of *Cichorium intybus* L. seed extract on renal parameters in experimentally induced early and late diabetes type 2 in rats. Ren Fail 39:211–221. 10.1080/0886022X.2016.1256317 27846769 10.1080/0886022X.2016.1256317 PMC6014526

[36] Gorinstein S, Leontowicz H, Leontowicz M, Krzeminski R, Gralak M, Delgado-Licon E, Martinez Ayala AL, Katrich E, Trakhtenberg S (2005) Changes in plasma lipid and antioxidant activity in rats as a result of naringin and red grapefruit supplementation. J Agric Food Chem 53:3223–3228. 10.1021/jf058014h 15826081 10.1021/jf058014h

[37] Valcheva-Kuzmanova S, Kuzmanov K, Mihova V, Krasnaliev I, Borisova P, Belcheva A (2007) Antihyperlipidemic effect of *Aronia melanocarpa* fruit juice in rats fed a high-cholesterol diet. Plant Foods Hum Nutr 62:19–24. 10.1007/s11130-006-0036-2 17136466 10.1007/s11130-006-0036-2

[38] Longo A, Veiga GB, Cousen MIS, Karpinski C, Schneider A, Weber B, Bertoldi EG, Borges LR, Bertacco RTA (2021) Factors associated to serum paraoxonase 1 activity in patients with cardiovascular disease. Arch Endocrinol Metab 65:676–683. 10.20945/2359-3997000000354 33844899 10.20945/2359-3997000000354 PMC10065381

[39] Chielle EO, Bonfanti G, De Bona KS, Cargnelutti LOL, Bitencourt PERL, da Silva PSL, Campos MMAL, Moretto MB (2018) Rutin restores adenosine deaminase activity in serum and the liver and improves biochemical parameters in streptozotocin-induced diabetic rats. Rev Bras Plantas Med 18(1 suppl 1):273–278. 10.1590/1983-084X/15_189

[40] Antonioli L, Pacher P, Haskó G (2022) Adenosine and inflammation: it’s time to (re)solve the problem. Trends Pharmacol Sci 43:43–55. 10.1016/j.tips.2021.10.010 34776241 10.1016/j.tips.2021.10.010

[41] Rachdaoui N (2020) Insulin: the friend and the foe in the development of type 2 diabetes mellitus. Int J Mol Sci 21(5):1770. 10.3390/ijms21051770 32150819 10.3390/ijms21051770 PMC7084909

[42] Muhammad IF, Borné Y, Hedblad B, Nilsson PM, Persson M, Engström G (2016) Acute-phase proteins and incidence of diabetes: a population-based cohort study. Acta Diabetol 53:981–989. 10.1007/s00592-016-0903-8 27581604 10.1007/s00592-016-0903-8 PMC5114318

[43] Sun S, Liu Z, Lin M, Gao N, Wang X (2024) Polyphenols in health and food processing: antibacterial, anti-inflammatory, and antioxidant insights. Front Nutr 11:1456730. 10.3389/fnut.2024.1456730 39224187 10.3389/fnut.2024.1456730 PMC11366707

[44] Ahmed N, El-Fateh M, Diarra MS, Zhao X (2026) Dietary polyphenols as functional food bioactives: nuclear factor erythroid 2-related factor 2 (Nrf2)-mediated antioxidant and immunomodulatory mechanisms in combating bacterial infections. J Funct Foods 137:107165. 10.1016/j.jff.2026.107165

[45] Pannucci E, Spagnuolo L, De Gara L, Santi L, Dugo L (2023) Phenolic compounds as preventive and therapeutic agents in diabetes-related oxidative stress, inflammation, advanced glycation end-products production and insulin sensitivity. Discov Med 35:715–732. 10.24976/Discov.Med.202335178.68 37811611 10.24976/Discov.Med.202335178.68

[46] Norton L, Shannon C, Gastaldelli A, DeFronzo RA (2022) Insulin: the master regulator of glucose metabolism. Metabolism 129:155142. 10.1016/j.metabol.2022.155142 35066003 10.1016/j.metabol.2022.155142

[47] Ahmad I, de Souza Cardoso J, de Mello JE, Teixeira FC, Saraiva JT, Bona NP, Vizzotto M, de Souza LP, Domingues WB, Campos VF, Lencina CL, Spanevello RM, Tavares RG, de Aguiar MSS, Stefanello FM (2025) Protective effects of *Eugenia uniflora* red fruit on brain in a rat model of type 2 diabetes: mechanistic insights. Neurochem Res 50:335. 10.1007/s11064-025-04587-5 41134426 10.1007/s11064-025-04587-5

[48] Cozza A, Chinigò C, Filicetti E, Greco GI, Lappano R, Marinaro C, Muglia L, Soraci L, Corsonello A, Lattanzio F, Volpentesta M (2025) Effects of antidiabetic medications on the relationship between type 2 diabetes mellitus and cognitive impairment. Ageing Res Rev 112:102834. 10.1016/j.arr.2025.102834 40659290 10.1016/j.arr.2025.102834

[49] Kopp KO, Glotfelty EJ, Li Y, Greig NH (2022) Glucagon-like peptide-1 (GLP-1) receptor agonists and neuroinflammation: implications for neurodegenerative disease treatment. Pharmacol Res 186:106550. 10.1016/j.phrs.2022.106550 36372278 10.1016/j.phrs.2022.106550 PMC9712272

[50] Hasheminasabgorji E, Jha JC (2021) Dyslipidemia, diabetes and atherosclerosis: role of inflammation and ROS-redox-sensitive factors. Biomedicines 9:1602. 10.3390/biomedicines9111602 34829831 10.3390/biomedicines9111602 PMC8615779

[51] Yener MD, Çolak T, Özsoy ÖD, Eraldemir FC (2024) Alterations in catalase, superoxide dismutase, glutathione peroxidase and malondialdehyde levels in serum and liver tissue under stress conditions. J İst Fac Med 87:145–152. 10.26650/IUITFD.1387837

[52] Chen X, Xie N, Feng L, Huang Y, Wu Y, Zhu H, Tang J, Zhang Y (2025) Oxidative stress in diabetes mellitus and its complications: from pathophysiology to therapeutic strategies. Chin Med J 138:15–27. 10.1097/CM9.0000000000003230 39503316 10.1097/CM9.0000000000003230 PMC11717531

[53] Jalouli M, Rahman MA, Biswas P, Rahman H, Harrath AH, Lee I-S, Kang S, Choi J, Park MN, Kim B (2025) Targeting natural antioxidant polyphenols to protect neuroinflammation and neurodegenerative diseases: a comprehensive review. Front Pharmacol 16:1492517. 10.3389/fphar.2025.1492517 39981183 10.3389/fphar.2025.1492517 PMC11840759

[54] Banji OJF, Banji D, Makeen HA, Alqahtani SS, Alshahrani S (2022) Neuroinflammation: the role of anthocyanins as neuroprotectants. Curr Neuropharmacol 20:2156–2174. 10.2174/1570159X20666220119140835 35043761 10.2174/1570159X20666220119140835 PMC9886846

[55] Dutta S, Shah RB, Singhal S, Dutta SB, Bansal S, Sinha S, Haque M (2023) Metformin: a review of potential mechanism and therapeutic utility beyond diabetes. Drug Des Devel Ther 17:1907–1932. 10.2147/DDDT.S409373 37397787 10.2147/DDDT.S409373 PMC10312383

[56] Figueira I, Tavares L, Jardim C, Costa I, Terrasso AP, Almeida AF, Govers C, Mes JJ, Gardner R, Becker JD, McDougall GJ, Stewart D, Filipe A, Kim KS, Brites D, Brito C, Brito MA, Santos CN (2019) Blood–brain barrier transport and neuroprotective potential of blackberry-digested polyphenols: an in vitro study. Eur J Nutr 58:113–130. 10.1007/s00394-017-1576-y 29151137 10.1007/s00394-017-1576-y

[57] Dokken BB, Sloniger JA, Henriksen EJ (2005) Acute selective glycogen synthase kinase-3 inhibition enhances insulin signaling in prediabetic insulin-resistant rat skeletal muscle. Am J Physiol Endocrinol Metab 288:E1188–E94 15671078 10.1152/ajpendo.00547.2004

[58] Rains JL, Jain SK (2011) Oxidative stress, insulin signaling, and diabetes. Free Radic Biol Med 50:567–575. 10.1016/j.freeradbiomed.2010.12.006 21163346 10.1016/j.freeradbiomed.2010.12.006 PMC3557825

[59] Gu Z, Mu H, Shen H, Deng K, Liu D, Yang M, Zhang Y, Zhang W, Mai K (2019) High level of dietary soybean oil affects the glucose and lipid metabolism in large yellow croaker *Larimichthys crocea* through the insulin-mediated PI3K/AKT signaling pathway. Comp Biochem Physiol B 231:34–41. 10.1016/j.cbpb.2018.12.003 30772486 10.1016/j.cbpb.2018.12.003

[60] Li X, Jayachandran M, Xu B (2021) Antidiabetic effect of konjac glucomannan via insulin signaling pathway regulation in high-fat diet and streptozotocin-induced diabetic rats. Food Res Int 149:110664. 10.1016/j.foodres.2021.110664 34600666 10.1016/j.foodres.2021.110664

